# The Korean herbal medicine, Do In Seung Gi-Tang, attenuates atherosclerosis via AMPK in high-fat diet-induced ApoE^−/−^ mice

**DOI:** 10.1186/s12906-016-1309-4

**Published:** 2016-09-08

**Authors:** Sun Haeng Park, Yoon-Young Sung, Seol Jang, Kyoung Jin Nho, Go Ya Choi, Ho Kyoung Kim

**Affiliations:** 1 Mibyeong Research Center, Korea Institute of Oriental Medicine, 1672 Yuseong-daero, Yuseong-gu, Daejeon 305-811 South Korea; 2 Department of Korean Medical Science, School of Korean Medicine, Pusan National University, Gyeongnam, Yangsan 626-870 South Korea; 3 K-herb Research Center, Korea Institute of Oriental Medicine, 1672 Yuseong-daero, Yuseong-gu, Daejeon 305-811 South Korea

**Keywords:** Atherosclerosis, Apolipoprotein E-deficient mice, Adenosine monophosphate-activated protein kinase, Do In Seung Gi-Tangm, Vascular inflammation

## Abstract

**Background:**

Do In Seung Gi-Tang (DISGT) is an herbal mixture of traditional Korean medicine that is composed of *Rheum undulatum* Linne, *Prunus Persica* (L.) Batsch, *Conyza canadensis* L., *Cinnamomum Cassia* Presl, and *Glycytthiza uralensis* Fischer (8: 6: 4: 4: 4 ratio). We investigated the effect of DISGT on vascular inflammation and lipid accumulation in apolipoprotein E-deficient (ApoE^−/−^) mice.

**Methods:**

ApoE^−/−^ mice that were fed a high-fat diet (HFD) were treated with DISGT (300 mg/kg/day) or statin (10 mg/kg/day) for 16 weeks. Serum lipid levels were analyzed. Oil Red O staining was used to evaluate atherosclerotic lesions and lipid accumulation in the aorta and liver, respectively. The expression of adhesion molecules (intercellular adhesion molecule-1 [ICAM-1], vascular cell adhesion molecule-1 [VCAM-1], and E-selectin), fatty acid synthase (FAS), adenosine monophosphate-activated protein kinase (AMPK), and acetyl-coA carboxylase (ACC) in the aorta or liver tissues was measured by western blot analysis. Lipid synthesis and inflammatory responses were assessed by immunohistochemistry and hematoxylin & eosin staining, respectively.

**Results:**

Treatment of HFD-fed mice with DISGT significantly lowered body weight, liver weight, and the levels of lipids, including total cholesterol, low-density lipoprotein-cholesterol, and triglycerides. Glucose levels were also lowered. In the aorta, DISGT attenuated atherosclerotic lesions and reduced the expression of ICAM-1, VCAM-1, and E-selectin. Moreover, DISGT decreased lipid accumulation, inflammatory responses, and FAS levels, and it activated AMPK and reduced ACC expression in liver tissues.

**Conclusions:**

The beneficial, anti-lipolytic, and anti-inflammatory effects of DISGT were mediated by the AMPK pathway. As a result, the expression of inflammatory factors was reduced. Our data provide evidence that DISGT may have strong therapeutic potential in treating vascular diseases, such as atherosclerosis.

## Background

Do In Seung Gi-Tang (DISGT; Tao He Cheng Qi-Tang; Chinese) is used in traditional Korean medicine to treat blood stasis by promoting blood circulation. Blood extravasation in traditional Korean medicine refers to the ability to encompass vascular diseases that are related to hyperlipidemia, such as arteriosclerosis [1–5]. DISGT has been shown to improve the impairment of acetylcholine-induced vasorelaxation responses by inducing endothelial nitric oxide synthase expression, thus reducing the accumulation of atherosclerotic lesions in vivo [2]. As documented in *Shang Han Lun*, DISGT is five species composed of *Rheum undulatum* Linne, *Prunus Persica* (L.) Batsch, *Conyza canadensis* L., *Cinnamomum Cassia* Presl, and *Glycytthiza uralensis* Fischer, that have various pharmacological on the cardiovascular disease or metabolic diaseas [1, 6, 7]. It has been reported to high-fat/high-cholesterol diet-fed *db/db* mice and western diet fed ApoE mice, DISGT decreased plasma glucose, cholesterol, triglyceride, and LDL cholesterol levels and [8]. Further, DISGT treat chronic hepatitis and amenorrhea. However, the mechanisms involved and effectiveness of DISGT remain unclear in atherosclerosis.

Atherosclerosis is a chronic inflammatory disease that involves increased total cholesterol (T-chole) levels, blood monocyte recruitment, lipid deposition, and macrophage foam cell formation [9, 10]. Recently, the atherosclero-related disease protective potentive of DISGT is suggest based on some studies. DISGT, has been shown to attenuate the increases in T-chole that are induced by a hypercholesterolemic diet in mices, and this action may be due to the promotion of cholesterol [1, 7]. These results show that DISGT may be useful in the treatment of cardiovascular-metabolic disease. The effects of adenosine monophosphate-activated protein kinase (AMPK) on metabolically relevant organs and tissues, such as the liver, skeletal muscle, adipose tissue, and hypothalamus, are relatively well documented [11]. The notion that AMPK activation could be used to promote vascular health has only recently emerged. In vascular tissues, AMPK activation appears to be a shared molecular target [12, 13]. Therefore, AMPK activation may improve vascular diseases, including atherosclerosis.

Traditional Korean herbal medicine has been widely used in the treatment of atherosclerosis-related disorders [14]. However, its therapeutic efficacies and mechanisms of action are unclear. Several studies have suggested that DISGT demonstrates therapeutic potential in cardiovascular disease and atherosclerosis. In this study, we investigated the effects of DISGT on atherosclerosis-related markers, including intercellular adhesion molecule-1 (ICAM-1), vascular cell adhesion molecule-1 (VCAM-1), and E-selectin, in high-fat diet (HFD)-induced apolipoprotein E-deficient (ApoE^−/−^) mice. In addition, we assessed the effect of DISGT on AMPK signaling, lipid levels, atherosclerotic lesions, and liver histology in these mice.

## Methods

### Preparation of DISGT (Table 1)

The herbs were purchased from Kwangmyeongdang Medicinal Herbs Co. (Ulsan, Korea) and authenticated based on their microscopic and macroscopic characteristics by the classification and identification committee of the Korea Institute of Oriental Medicine (KIOM).

**Table 1:** Composition of Do In Seung Gi-Tang

Scientific name	Herbal name	Amount (g)
*Prunus persica* (L.) Batsch	Persicae Semen, 桃仁	60
*Cinnamomum cassia* Presl	Cinnamomi Ramulus, 桂枝	40
*Glycyrrhiza uralensis* Fisch	Licorice, 甘草	40
*Mirabilite*	Natrii sulfas, 芒硝	40
*Rheum palmatum* Linne	Rhei Rhizoma, 大黃	80

The formula of DIGST consists of five herbs, including *Rheum undulatum* Linne (80 g), *Prunus Persica* (L.) Batsch (60 g), *Conyza canadensis* L. (40 g), *Cinnamomum Cassia* Presl (40 g), and *Glycyrrhiza uralensis* Fischer (40 g), which were mixed and ground into a crude powder. The total mixure (260 g) was boiled in distilled water (1:10, v/v) at 100 °C for 2 h and the extract was filtered, lyophilized and subsequently stored at −20 °C. The yield of DISGT aqueous extract was 11.25 % (w/w).

### Experimental animals, diet, and treatments

Male 8 weeks old *ApoE*^−/−^ mice on a C57BL/6 J background were obtained from SLC Inc., (Shizuoka, Japan). They were housed under diurnal lighting conditions and allowed access to food and tap water *ad libitum*. All experimental protocols involving the use of animals were conducted in accordance with the National Institutes of Health guidelines and approved (approve number; Kiom 13–037) by the Committee on Animal Care of the Korea Institute of Oriental Medicine (KIOM).

Mice were divided into four groups: (1) Normal diet (ND; *n* = 8), (2) HFD (41 % of total calories from fat; 0.21 % cholesterol; Research Diet, New Brunswick, NJ, USA) with saline (*n* = 8), (3) HFD with 300 mg/kg DISGT (*n* = 8), and (4) HFD with 10 mg/kg atorvastatin (statin; *n* = 8). DISGT or statin was dissolved in water and then given orally everyday over a 16-week period, during which the mice were fed a HFD. Control mice received saline.

### Analysis of serum markers

Blood samples were drawn from the aorta under light anesthesia and stored on ice for 30 min before centrifugation at 13,000 rpm at 4 °C for 10 min, after which they were stored at −80 °C. Serum levels of T-chole, high-density lipoprotein cholesterol (HDL), low-density lipoprotein cholesterol (LDL), triglyceride (TG), glucose, alanine aminotransferase (ALT), aspartate aminotransferase (AST), and creatinine were measured with an automatic analyzer (Hitachi Co., Tokyo, Japan).

### Atherosclerotic lesion analysis

Mice were anesthetized and euthanized after 16 weeks of HFD or ND. The aortas were washed with phosphate-buffered saline (PBS) and then fixed with 10 % paraformaldehyde overnight. The adventitia was cleaned thoroughly under a dissecting microscope, and fixed aortas were stained with Oil Red O stock solution (0.3 %) for 1 h. Atherosclerotic lesion areas were quantified using the isolution full-image analysis software (Image and Microscope Technology, Vancouver, Canada).

### Histopathology

Mice were deeply anesthetized with sodium thiopental and subsequently perfused with cold PBS, followed by fixation with 10 % (w/v) paraformaldehyde overnight. *Hematoxylin and Eosin (H&E)*: Livers were embedded in paraffin and sectioned serially at 4 μm. The sections were stained with H&E. The sections were stained with H&E as described previously [15]. *Immunohistochemistry*: Livers were removed and fixed in the same solution for 24 h at 4 °C. Fixed aortas were frozen and stored at −80 °C. Sections that were 10-μm thick were sliced from frozen tissues and then immunostained with antibodies against fatty acid synthase (FAS; Novus Biologicals; Littleton, CO, USA). After additional incubation with the secondary antibody, the chromogen, 3-amino-9-ethylcarbazole, was added to the sections (Vector Laboratories; Burlingame, CA, USA). Reactions with 3,3’-diaminobenzidine substrate (Vector Laboratories) were performed for color development, and the stained sections were then analyzed by light microscopy scanner (3D HISTECH, Budapest, Hungary).

### Western blot analysis

Aortas were homogenized in Tissue protein extraction reagent (Thermo Scientific; Pittsburgh, PA, USA), which was supplemented with a 1 % phosphatase inhibitor cocktail (Sigma-Aldrich Chemical Co.; St. Louis, MO, USA) and protease inhibitors (Roche Applied Science; Basel, Switzerland). The recovered proteins were isolated from aortic tissues, separated by 10 % sodium dodecyl sulfate-polyacrylamide gel electrophoresis, and then transferred onto nitrocellulose membranes (Amersham Biosciences; Piscataway, NJ, USA), according to standard techniques. Blots were probed with primary antibodies directed against VCAM-1 (Santa Cruz Biotechnology; Santa Cruz, CA, USA), ICAM-1, E-selectin, AMPK, phosphorylated (p)-AMPK, acetyl-COA carboxylase (ACC), p-ACC (Cell Signaling Technology; Beverly, MA, USA), or FAS. This was followed by incubation with the secondary antibody conjugated to horseradish peroxidase (Cell Signaling). Bound secondary antibodies were detected by chemiluminescence, as generated by the peroxidase reaction, and they were measured with an ImageQuant LAS 4000 apparatus (GE Healthcare Life Sciences; Buckinghamshire, UK). The membranes were then reprobed with an anti-β-actin antibody (Cell Signaling) as the internal sample control.

### Quantitative high-performance liquid chropmatography analysis

The sample was analyzed by reverse phase-high performance liquid chromatography using Waters e2695 liquid chromatography system (Waters Co., Milford, MA, USA), equipped with a Waters 2998 photodiode array detector. Data processing was carried out with the Empower software (Waters Co.). The Phenomenex Luna C18 column (250 mm × 4.6 mm; particle size 5 μm; Phenomenex, Torrance, CA, USA) was used as the stationary phase and 1 % (v/v) acetic acid aqueous solution (A) and acetonitrile · acetic acid (99:1) (B) were used as the mobile phase. The elution conditions involved holding the starting mobile phase at 90 % A and 10 % B and applying a gradient of 0 % A and 100 % B for 50 min. A wash with 100 % acetonitrile was applied for 10 min, followed by equilibration at 90 % A and 10 % B for 10 min. The flow rate was 1.0 mL/min and the injection volume for all the samples was 20 μL. Peaks were identified by comparing retention times and UV spectra with those of commercial standards. Components were quantified based on peak areas at 220 nm. Quantitation was performed based on a mixture of external standards of known concentration, which were analyzed in duplicate before and after analyzing the samples. Peak areas were used to calculate the quantities of compounds in the samples. The calibration curves of the standards ranging from 11.9 to 243 μg/mL (five levels) revealed good linearity, with R^2^ values exceeding 0.999 (peak area vs. concentration). HPLC-grade reagents, acetonitrile and water were obtained from J. T. Baker (Phillipsburg, NJ, USA).

### Data analysis

Values are expressed as the mean ± standard error of the mean (SEM). Statistical comparisons were performed using unpaired Student’s t-tests and analysis of variance (ANOVA) for repeated measures followed by post hoc pairwise comparisons in SigmaPlot software version 13.0 (Systat Software Inc.; San Jose, CA, USA). Differences were considered significant at *p* < 0.05.

## Results

### Effect of DISGT on body weight, liver weight, and serum parameters in HFD-induced ApoE^−/−^ mice

**Fig. 1:**
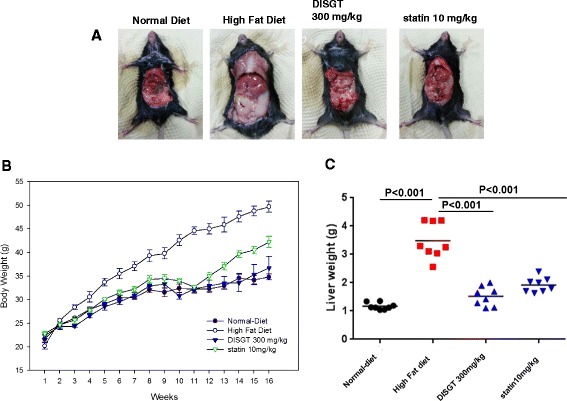
Effects of DISGT on body weights and liver weights. Mice (8 weeks old) were placed in four groups: normal diet (ND), high-fat diet (HFD), HFD with 300 mg/kg Do In Seung Gi-Tang (DISGT), and HFD with 10 mg/kg atorvastatin (statin). Experiments were carried out for 16 weeks. **a** Images of mice from all groups. **b** Body weights were measured every week for 16 weeks. **c** After 16 weeks of ND or HFD, liver tissues were collected and washed with cold phosphate-buffered solution, after which they were weighed. Values are mean ± SEM. **p < 0.01, ***p < 0.001 vs. ND; ^*###*^ *p <* 0.001 vs. HFD

The body weight, liver weight, and serum parameters were measured in mice. HFD-fed ApoE^−/−^ mice showed significantly increased body weight compared with ND-fed mice (34.8 ± 0.59 vs. 49.7 ± 1.16 g, *p <* 0.001). However, treatment of HFD-fed mice with DISGT or statin decreased the body weight (Fig. 1a-b and Table 2; 36.7 ± 2.48 or 42.2 ± 1.24 g vs. HFD group; *p <* 0.001). Furthermore, the liver weights showed an increased in the HFD-fed ApoE^−/−^ mice (*p* < 0.001), and the elevated liver weight was reduced by DISGT (Fig. 1c and Table 2; *p* < 0.001, versus HFD group). In addition, HFD-fed mice showed significantly higher serum levels of T-chole, LDL, TGs, glucose, AST, ALT, and creatinine than that of ND-fed mice. These levels were all significantly reduced by DISGT or statin treatment (Table 3).

**Table 2:** Body weight and liver weight

			DISGT	Statin
	ND	HFD	300 mg/kg	10 mg/kg
Body weight (g)				
Initial body weight	21.70 ± 0.64	20.20 ± 0.67	22.40 ± 0.57	22.80 ± 0.50
Final body weight	34.80 ± 0.59	49.70 ± 1.16***	36.70 ± 2.48^###^	42.20 ± 1.24^###^
Body weight gain	13.10 ± 0.63	29.50 ± 0.98***	14.30 ± 1.00^###^	19.40 ± 0.65^###^
Liver weight (g)	1.17 ± 0.05	3.48 ± 0.24***	1.52 ± 0.13^###^	1.92 ± 0.10^###^

*Abbreviations*: *HFD* high-fat diet, *DISGT* Do In Seung Gi-Tang

All data represent mean values ± SEM (*n* = 8)

^***^ *p* < 0.001, vs. *ApoE* ^*−*/−^ mice without HFD (ND; Normal diet)

^###^ *p* < 0.001, vs. HFD-fed *ApoE* ^−/−^ mice (HFD)

**Table 3:** Lipid parameters

			DISGT	Statin
	ND	HFD	300 mg/kg	10 mg/kg
T-chole (mg/dL)	401.86 ± 21.18	1401.71 ± 69.66***	951.43 ± 56.72^###^	1145.00 ± 75.60^#^
LDL (mg/dL)	71.86 ± 2.08	368.29 ± 17.88***	244.14 ± 13.48^###^	316.33 ± 18.48^#^
HDL (mg/dL)	22.14 ± 1.27	11.71 ± 1.14***	12.57 ± 1.15	13.17 ± 2.71
TG (mg/dL)	34.57 ± 2.51	278.71 ± 16.06***	43.29 ± 13.11^###^	75.83 ± 2.42^###^
Glucose (mg/dL)	134.29 ± 4.40	422.71 ± 36.80***	201.29 ± 12.79^###^	298.50 ± 12.83^##^
AST (U/L)	50.29 ± 7.06	571.57 ± 22.64***	333.29 ± 25.70^###^	370.83 ± 12.62^###^
ALT (U/L)	29.86 ± 1.71	562.43 ± 14.32***	247.86 ± 33.41^###^	277.00 ± 20.91^###^
Creatinine (mg/dL)	0.21 ± 0.02	0.35 ± 0.03**	0.17 ± 0.02^###^	0.25 ± 0.03

*Abbreviations*: *HFD* high-fat diet, *DISGT* Do In Seung Gi-Tang, *T-chole* total cholesterol, *LDL* low-density lipoprotein, *HDL* high-density lipoprotein, *TG* triglyceride, *AST* aspartate aminotransferase, *ALT* alanine aminotransferase

All data represent mean values ± SEM (*n* = 8)

^**^ *p* < 0.01, ^***^ *p* < 0.001, versus *ApoE* ^−/−^ mice without HFD (ND)

^#^ *p* < 0.05, ^##^ *p* < 0.01, and ^###^ *p* < 0.001, versus HFD-fed *ApoE* ^−/−^ mice (HFD)

### Effect of DISGT on atherosclerotic lesions in aortas

**Fig. 2:**
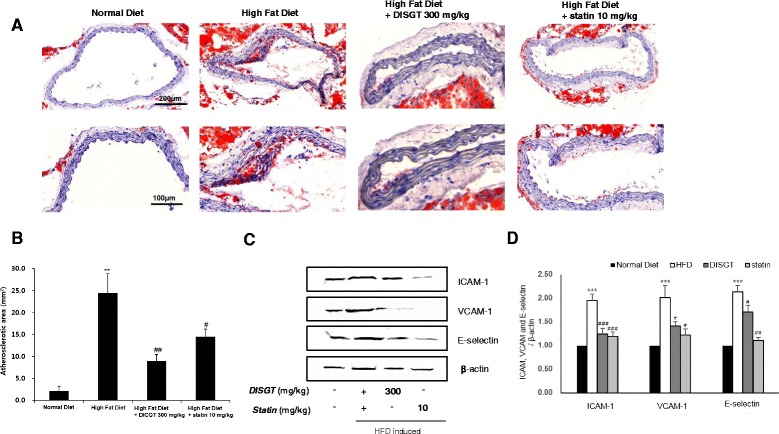
Effect of DISGT on reducing aortic lesions and expression of adhesion molecules in the aorta. **a** The aorta was collected from HFD-fed mice that were treated with DISGT or statin orally for 16 weeks. Representative photomicrographs of Oil Red O staining are shown. (Cross sections; ×100, above; ×200, under, magnification). **b** Quantification of Oil Red O-stained atherosclerotic lesion areas from each group. **c** Western blots of ICAM-1, VCAM-1, and E-selectin. **d** Densitometric analysis showing the relative protein expression levels of ICAM-1, VCAM-1, and E-selectin that were normalized to β-actin. Values are mean ± SEM. **p < 0.01, ***p < 0.001 vs. ND; ^#^ *p* < 0.05, ^##^ *p* < 0.01, ^###^ *p* < 0.001vs. HFD

The extent of atherosclerotic lesions in the aorta was determined by isolution analysis, and it was confined to the cross-section of the aortic root. Oli Red O staining was used to detect atherosclerotic plaques. The aortic root lesion area was markedly increased in HFD-fed mice (27.72 ± 2.87 mm^2^; *p* < 0.01), and it was significantly reduced by treatment with DISGT or statin (7.24 ± 1.81 mm^2^ or 11.90 ± 1.07 mm^2^; *p* < 0.01) (Fig. 2a and b). As demonstrated by western blot analysis, the expression of ICAM-1, VCAM-1, and E-selectin in HFD-fed mice was significantly increased compared with that in ND-fed mice. In contrast, DISGT- or statin-treated mice showed lower expression levels of ICAM-1, VCAM-1, and E-selectin compared with HFD-fed mice (Fig. 2c), and this decrease by DISGT was statistically significant (Fig. 2d).

### Histology of liver

**Fig. 3:**
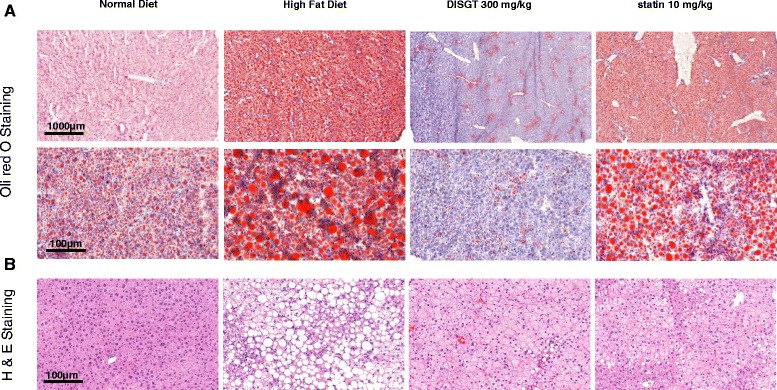
Effect of DISGT on ameliorating liver steatosis in HFD-fed ApoE^−/−^ mice. Liver tissues were fixed in 10 % formaldehyde, embedded in paraffin, sectioned, and stained with (**a**) Oil Red O (×20, above; ×100, under, magnification) or (**b**) Hematoxylin and eosin (×100 magnification)

The accumulation of lipids in the liver impairs hepatocyte function, which leads to hyperglycemia via hepatic glucose production. The staining of liver tissues with H&E and Oil Red O was analyzed. Lipid droplets accumulated in the livers of HFD-fed mice, and this accumulation was decreased with DISGT or statin treatment (Fig. 3a). Furthermore, DISGT reduced sizes of adipocytesation and inflammation, as demonstrated by decreased H&E staining (Fig. 3b).

### Effect of DISGT on FAS expression

**Fig. 4:**
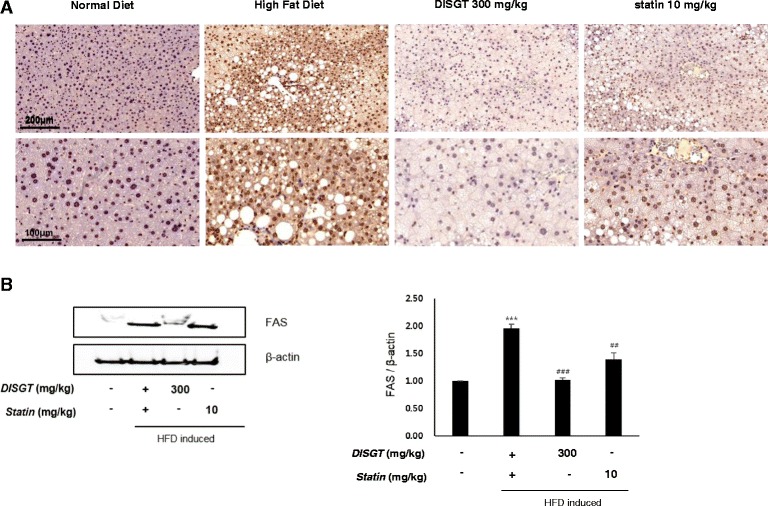
Effect of DISGT on attenuating fatty acid synthase (FAS) expression in the livers of HFD-fed ApoE^−/−^ mice. **a** Expression of lipogenic enzymes, such as FAS, and inflammatory factors in the livers and aortas of HFD-fed mice, respectively. **b** Protein expression levels of FAS were determined by western blot analysis. Values are mean ± SEM. ***p < 0.001 vs. ND; ^##^ *p* < 0.01, ^###^ *p* < 0.001vs. HFD

FAS is involved in the synthesis of fatty acids and TGs, and it is known to be upregulated in HFD-fed mice. The expression of FAS in liver tissues of HFD-fed mice was reduced by DISGT or statin treatment (Fig. 4a). Also, the effect of DISGT on decreasing FAS expression was further confirmed by western blotting (Fig. 4b).

### Effect of DISGT on the expression of the AMPK pathway in liver tissues

**Fig. 5:**
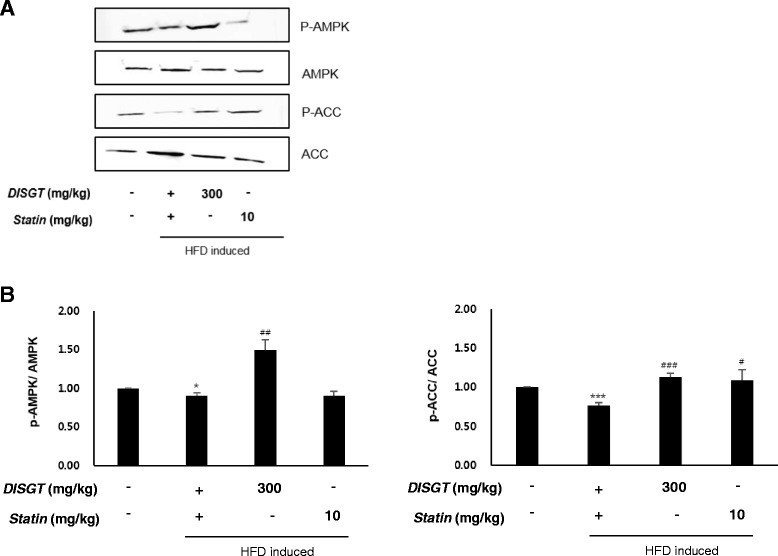
Effect of DISGT on adenosine monophosphate-activated protein kinase (AMPK) signaling pathway components. **a** Western blots of phosphorylated AMPK (p-AMPK), phosphorylated acetyl-COA carboxylase (p-ACC), AMPK, and ACC. **b** Densitometric analysis showing the relative protein expression levels of p-AMPK and p-ACC that were normalized to total AMPK and total ACC, respectively. Values are mean ± SEM. Values are mean ± SEM. *p < 0.05, ***p < 0.001 vs. ND; ^#^ *p* < 0.05, ^##^ *p* < 0.01, ^###^ *p* < 0.001vs. HFD

AMPK plays a crucial role in fatty acid metabolism, and it has demonstrated potential therapeutic impact in atherosclerosis. The protein levels of AMPK and ACC were assessed in liver tissues of ApoE^−/−^ mice. Interestingly, DISGT increased the phosphorylation of AMPK in liver tissues. ACC phosphorylation, which is an index of AMPK activation, was also consistently detected in these tissues (Fig. 5a and b).

### Quantitative high-performance liquid chropmatography analysis of DISGT

**Fig. 6:**
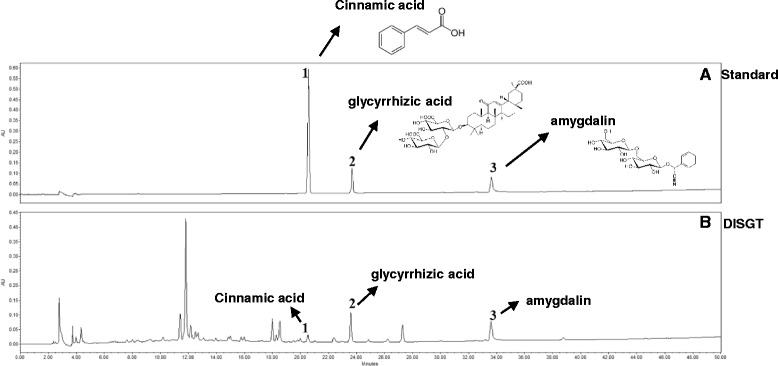
HPLC chromatogram of a standard mixture (**a**) and DISGT (**b**) at 220 nm. Cinnamic acid (1), glycyrrhizic acid (2), and amygdalin (3) appeared with retention times of approximately 20.5 min, 23.0 min, and 33.5 min, respectively

The HPLC analysis of the DISGT revealed three peaks matching those of the commercial standards cinnamic acid, glycyrrhizic acid, and amygdalin with retention times of approximately 20.5 min, 23.0 min, and 33.5 min, respectively (Fig. 6). The DISGT contained 0.104 ± 0.006 mg/g cinnamic acid, 1.901 ± 0.013 mg/g glycyrrhizic acid, and 2.566 ± 0.007 mg/g amygdalin (Table 4).

**Table 4:** Average contents of the reference compounds in DISGT

Compound	Average content (mg/g)
Cinnamic acid	0.104 ± 0.006
Glycyrrhizic acid	1.901 ± 0.013
Amygdalin	2.566 ± 0.007

## Discussion

The Korean herbal medicine DISGT, has been shown to prevent vascular or liver inflammatory responses and lipid deposition in vivo. Currently, it is used to treat blood stasis by promoting blood circulation. In this study, we evaluated the anti-atherosclerotic properties of DISGT.

Our findings show that DISGT reduced body weight, liver weight, and the serum levels of T-chole, LDL, TGs, and glucose, in HFD-fed ApoE^−/−^ mice. No changes in HDL were observed. DISGT also significantly attenuated the severity of atherosclerotic lesions and reduced the expression of adhesion molecules in the aortas of HFD-fed mice. In addition, the increased accumulation of inflammatory cells and hepatic steatosis in the liver tissues were observed in the HFD group compared with the ND group. However, DISGT inhibited these changes in HFD-fed mice. Importantly, the AMPK pathway is a regulator of metabolic homeostasis. DISGT markedly reduced the expression of FAS and p-ACC and increased the expression of p-AMPK. These data suggest that DISGT activates AMPK to exert anti-atherosclerotic effects.

Because AMPK is a cellular energy sensor, understanding the mechanism by which hepatic AMPK coordinates hepatic energy metabolism is important. The dysfunction of the AMPK signaling pathway is involved in the development of various cardiovascular diseases, including atherosclerosis [9, 16]. AMPK inhibits the expression of adhesion molecules and E-selectin in endothelial cells that have been exposed to hydrogen peroxide, tumor necrosis factor-alpha, or fatty acids [12, 17, 18]. In the liver, AMPK activation stimulates fatty acid oxidation and inhibits lipogenesis, and lipid metabolism is reduced by adiponectin [17, 18, 19]. Recent studies have suggested that AMPK is a therapeutic target of atherosclerosis, because it reduces vascular inflammation and fatty acid synthesis to attenuate atherosclerosis [13, 18, 20].

Many studies have focused on the effects of dietary fatty acids or vascular inflammation on lipid and lipoprotein metabolism with regard to the risk of atherosclerosis. However, fatty acids can also influence a number of other relevant mechanisms that are involved in atherosclerosis, such as lipid peroxidation, vascular inflammation, and hemostasis [21–23]. In our study, DISGT attenuated the accumulation of lipids in the liver and the presence of atherosclerotic lesions in the aorta. Furthermore, the expression levels of AMPK-regulated lipoxidation factors, inflammatory compounds, and adhesion molecules were significantly decreased in the livers and aortas of DISGT-treated, HFD-fed mice. This indicates that DISGT activates AMPK, thereby preventing the vascular inflammatory response and lipid deposition.

Interestingly, statins did not lower total cholesterol but had a major effect on TG levels in the current study. These results are different than those expected in humans. Clinically established statins have long been known to exert anti-atherosclerotic effects. However, recent studies have suggested that the effects of statins on cholesterol in mice are different than those in humans. For instance, statin treatment does not decrease cholesterol levels in ApoE^−/−^ mice. This can be explained by the lack of ApoE, which is a ligand for the LDL receptor and is necessary for LDL clearance. Thus, the cholesterol-independent effects of statins can be more effectively studied in mice than in humans [23–25].

Cholesterol crystals have been reported to be the metabolic signals that trigger sterile inflammation in atherosclerosis [26]. Previous studies have shown that AMPK is a key player in maintaining physiological processes in the heart and vasculature [12, 27], and it regulates lipoxidation genes [28]. Results from these studies support our data and suggest that AMPK modulates the effect of DISGT on lowering inflammation and lipid deposition to reduce atherosclerosis. Most importantly, DISGT protected the vasculature against the initiation and development of atherosclerosis.

## Conclusions

DISGT prevented the development of atherosclerotic lesions, the expression of vascular inflammatory markers, and the accumulation of lipids in HFD-fed ApoE^−/−^ mice. This was associated with increases in AMPK levels. Therefore, DISGT may exert its anti-atherosclerotic effects by activating AMPK. Moreover, DISGT has the potential for use as a therapeutic drug for the treatment of atherosclerosis. Further studies are needed to identify and classify the active components of the herbal mixture and clinical trial.

## Abbreviations

ACC: Acetyl-COA carboxylase
AMPK: Adenosine monophosphate-activated protein kinase
ApoE: Apolipoprotein E
FAS: Fatty acid synthase
HDL: High-density lipoprotein
ICAM: Intercellular adhesion molecule
KIOM: Korea Institute of Oriental Medicine
LDL: Low-density lipoprotein
T-chole: Total cholesterol
VCAM: Vascular cell adhesion molecule
